# Dominant carnivore loss benefits native avian and invasive mammalian scavengers

**DOI:** 10.1098/rspb.2022.0521

**Published:** 2022-10-26

**Authors:** Matthew W. Fielding, Calum X. Cunningham, Jessie C. Buettel, Dejan Stojanovic, Luke A. Yates, Menna E. Jones, Barry W. Brook

**Affiliations:** ^1^ School of Natural Sciences, University of Tasmania, Sandy Bay, Tasmania 7001, Australia; ^2^ ARC Centre of Excellence for Australian Biodiversity and Heritage, Sandy Bay, Tasmania 7001, Australia; ^3^ Fenner School of Environment and Society, Australian National University, Canberra, Australia; ^4^ School of Environmental and Forest Sciences, College of the Environment, University of Washington, Seattle, WA 98195-2100, USA

**Keywords:** scavenger, trophic cascade, carcass use, survival analysis, mesoscavenger release, carnivore extinction

## Abstract

Scavenging by large carnivores is integral for ecosystem functioning by limiting the build-up of carrion and facilitating widespread energy flows. However, top carnivores have declined across the world, triggering trophic shifts within ecosystems. Here, we compare findings from previous work on predator decline against areas with recent native mammalian carnivore loss. Specifically, we investigate top-down control on utilization of experimentally placed carcasses by two mesoscavengers—the invasive feral cat and native forest raven. Ravens profited most from carnivore loss, scavenging for five times longer in the absence of native mammalian carnivores. Cats scavenged on half of all carcasses in the region without dominant native carnivores. This was eight times more than in areas where other carnivores were at high densities. All carcasses persisted longer than the three-week monitoring period in the absence of native mammalian carnivores, while in areas with high carnivore abundance, all carcasses were fully consumed. Our results reveal that top-carnivore loss amplifies impacts associated with carnivore decline—increased carcass persistence and carrion access for smaller scavengers. This suggests that even at low densities, native mammalian carnivores can fulfil their ecological functions, demonstrating the significance of global carnivore conservation and supporting management approaches, such as trophic rewilding.

## 1. Introduction

Scavenging is ubiquitous among mammalian and avian carnivores, with most species scavenging to some degree [1–4]. Larger carnivorous mammals are highly efficient scavengers, consuming carcasses faster than most other taxa [5]. However, many larger mammalian carnivores, as well as obligate scavengers like vultures, are experiencing widespread declines due to habitat loss, disturbance and persecution by humans [6,7]. Fluctuations in the abundance of these species can have trophic consequences that cascade throughout the food web and impact nutrient cycling and increase the risk of disease transmission [8–10]. With populations of some larger mammalian carnivores now beginning to recover, while others continue to decline, this raises questions about how scavenging dynamics have shifted within modified ecosystems [11,12].

Larger mammalian carnivores can either provision ecosystems with a more stable supply of carrion (e.g. wolves in Yellowstone National Park [13]), or limit carrion access (e.g. bears kleptoparasiting cougar kills [14] and Tasmanian devils reducing carrion availability [15]). Carrion is a high-quality resource with low handling costs, and thus is attractive to mesoscavengers [1]. However, scavenging on carrion is also risky due to the increased likelihood of encountering dominant scavengers [16–18]. These competitive and facilitative processes can potentially make carrion ‘fatally attractive' for mesoscavengers [19]. For example, mesoscavengers were attracted to wolf kills yet were negatively associated with wolf density at the landscape scale [20]. Although carcasses are attractive to mesoscavengers, avoidance of dominant predators plays an important role in shaping carnivore communities [19,21].

**Figure 1.**
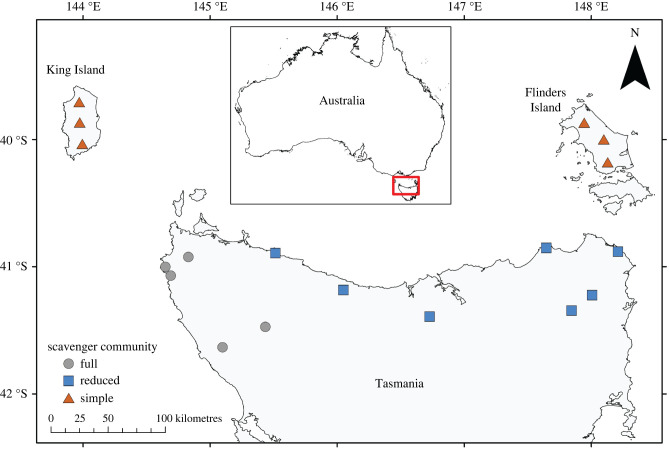
Geographical location of study sites across northern Tasmania and the Bass Strait Islands. Each shape indicates a site that contains between six and eight camera traps. (Online version in colour.)

Across the southern-temperate continental island of Tasmania (Australia) and its large offshore islands (figure 1; total area: 68 401 km^2^), a large-scale natural experiment is occurring due to the severe population decline of the largest extant terrestrial carnivore, the marsupial Tasmanian devil *Sarcophilus harrisii* [21]. Devils are Tasmania's dominant scavenger, being both the largest extant terrestrial mammalian carnivore and a specialist, although facultative, scavenger adapted for processing the toughest parts of carcasses [22]. Devils have experienced severe population declines due to a transmissible cancer, devil facial tumour disease (DFTD) [23]. The disease has progressively spread across Tasmania over 25 years, causing average population declines of 83% across approximately 90% of Tasmania [24,25]. The progressive spread of DFTD has created a natural experiment because regions of Tasmania have different disease histories and consequently, widely variable densities of top carnivores. Unlike most threatened carnivores [6], devil population declines are not caused by humans, allowing us to study the effects of a carnivore's abundance with little anthropogenic confounding [21]. In areas where devils have declined, carrion persists threefold longer, allowing increased carrion consumption by native (spotted-tailed quolls *Dasyurus maculatus*) and invasive (feral cats *Felis catus*) mammalian and avian (forest ravens *Corvus tasmanicus*) mesoscavengers [15]. However, this prompts the question: what would happen to carrion if all native mammalian carnivores were extirpated? Can invasive and avian mesoscavengers fully replace the ecosystem services of larger mammalian scavengers?

The Bass Strait Islands, between Tasmania and the Australian mainland, are ecologically similar to mainland Tasmania due to intermittent land connectedness during glacial maxima [26]. However, following major land-use change and human persecution, several species were driven to extinction on the islands, including mammalian carnivores like spotted-tailed quolls [27,28]. While there is no evidence of the Tasmanian devil on the islands following European occupation, on Flinders Island, fossil evidence suggest that devils may have persisted until at least 8000 years ago [28,29]. This mensurative experiment on the Bass Strait Islands thereby provides a unique opportunity to compare scavenging between: (i) a full community (Tasmanian mainland) comprising a native mammalian apex scavenger, native mammalian mesoscavenger, invasive-mammalian mesoscavenger and native avian mesoscavenger; (ii) a community in which only the native apex scavenger has declined (Tasmanian mainland in diseased areas); and (iii) a community lacking all native mammalian scavengers (Bass Strait Islands).

In this study, we build upon a previous dataset which investigated the impacts of Tasmanian devil decline on scavenging dynamics [15]. Here, we used experimentally deployed carcasses and camera traps to monitor carrion use by mammalian and avian scavengers on the two largest Bass Strait Islands, an area where native mammalian carnivores—the Tasmanian devil and spotted-tailed quoll—have been recently extirpated. Under this design, we investigated how the complete loss of native top scavengers (devils) and native mesoscavengers (quolls) impacts: (i) carrion discovery and use by invasive and extant-native mesoscavengers, and (ii) carcass persistence within an environment. We also tested the effect of habitat (wet versus dry forest) on carcass use and persistence, as we hypothesized that carcasses decomposition would be higher in wet environments due to greater microbial activity. Previous work by Cunningham *et al*. [15] found that avian generalist mesoscavengers (ravens) and invasive mesoscavengers (feral cats) could not match the scavenging efficiency of devils, leading to increased carcasses persistence in areas where devils have declined but are still present. They also found that top native mammalian scavengers (devils) limit carrion access and total feeding time for other smaller scavengers. In our study system, devoid of both devils and quolls, we hypothesized both impacts on carcass persistence and carcass discovery and use would be exacerbated. Furthermore, we hypothesized that devils would have a stronger impact on the scavenging community than smaller native mesoscavengers (quolls).

## 2. Material and methods

### (a) Study area

We measured carrion use by mammalian and avian scavengers across Tasmania and the two largest Bass Strait Islands in southeastern Australia (figure 1). In Tasmania, the progressive westward spread of DFTD from its origin in the northeast, followed by rapid and severe local population decline has resulted in low devil densities across most of the state. We divided northern Tasmania and the Bass Strait islands into three regions, partitioned geographically based on the density of native mammalian scavengers: (i) a full community, where DFTD was absent or only recently invaded, and devils were abundant and quolls present although naturally at lower densities than devils; (ii) a reduced community, where DFTD was prevalent and devil numbers declined by more than 80%, however, quoll densities do not appear to have increased substantially; and (iii) a simple community, where devils and quolls are locally extinct (figure 1). We selected 18 study sites across the three regions (full community = 7; reduced community = 5; simple community = 6). As Tasmanian mainland sites were selected as part of an earlier study [15], sites on the Bass Strait Island were selected to be comparable (within forested areas with minimum human influence i.e. largely unsealed roads with low traffic and no recent logging). Prior to carcass placement, the community classifications were confirmed using remote-camera surveys to monitor devil and quoll activity (see electronic supplementary material).

For the scavenging surveys, we experimentally deployed 136 carcasses and cameras across these regions (full community, 40; reduced community, 56; simple community, 40) with six to eight carcasses per site. Sites were placed in a roughly even mixture of wet eucalypt/rainforest habitat and dry eucalypt/coastal scrub habitat and we selected areas where human influence was minimal. To ensure the study units were comparable, we selected sites with similar average annual rainfall (wet, 900–1800 mm; dry, 600–1200 mm) and mean maximum temperature in August (9–15°C) (Bureau of Meteorology; bom.gov.au). Following the study, we also ensured there were no climatic anomalies between the years of the study that might cause confounding effects.

### (b) Experimental design

Carcasses were deployed during August–September 2016 for the Tasmanian mainland, and August–September 2020 on the Bass Strait islands. To prevent the early removal of carcasses from the view of the camera, carcasses on the Tasmanian mainland were secured to the ground with a short stake. Carcasses on the Bass Strait islands were not secured as we expected that the carcasses were unlikely to be moved due to the lack of any large mammalian carnivores. We worked in late winter, when consumption by invertebrate scavengers and microbial decomposers is generally at its lowest. We used Bennett's wallaby (*Macropus rufogriseus*; 13.8–18.6 kg) and Tasmanian pademelon (*Thylogale billardierii*; 1.5–8 kg) carcasses. These are both regularly culled under crop-protection permits and are a common source of carrion in the study region. The carcass species used at a given site depended on local availability and the primary macropod species in the area (simple community: Bennett's wallaby = 38; Tasmanian pademelon = 2; reduced community: Tasmanian pademelon = 56; full community: Tasmanian pademelon = 40). To ensure independence, carcasses were deployed at least 1 km apart. At each carcass, we installed one camera trap (Cuddeback X-Change 1279 or Reconyx PC-800), programmed to take a photo when triggered by movement with a wait period of 3 min between photos. Cameras were deployed for a minimum of 21 days, after which we expected the carcasses to be mostly consumed by scavengers based on studies in similar settings [30].

### (c) Analysis

#### (i) Carcass discovery and persistence

All analyses were completed using R v4.0.2 [31]. We used statistical survival analysis based on a mixed-effects Cox proportional hazards model from the R package ‘coxme' [32] to study carcass discovery and persistence. We ran separate analyses to investigate the time it took for the carcasses to be discovered by (i) any vertebrate scavenger, (ii) ravens, and (iii) feral cats. Discovery was defined as the first time an animal found and fed on the carcass. Carcasses were defined as fully consumed when there was a clear final consumption event, and the physical carcass was absent from subsequent images. We used survival analysis because the discovery and persistence data were censored [33] (see electronic supplementary material for further methodology).

We tested a range of survival models with combinations of four predictor variables: devil activity (number of devil detections per 100 camera nights), quoll activity (number quoll detections per 100 camera nights) and habitat (wet versus dry forest), with initial carcass weight (kg) included as a covariate to account for variation in carcass size (see electronic supplementary material, table S1 for model combinations). To account for variation across the study sites, we used site location as a random effect. All the predictors had a Pearson's cross-correlation coefficient *r* < 0.7. We scored the models using estimates of the Kullback–Leibler discrepancy, which was calculated using leave-one-out cross validation (LOOCV) [34]. As the log partial likelihood of a mixed-effects Cox proportional hazards model is based on time-to-event data, ordinary LOOCV cannot be applied, as it requires at least two observations in the test set [35]. Therefore, we used an alternative method for cross validation in Cox models, in which the log partial likelihood for the training data are subtracted from the log partial likelihood of the entire dataset [36]. Models were ranked in complexity using the effective degrees of freedom (*p*_eff_), calculated by subtracting the cross-validated log-likelihood from the within-sample log-likelihood, which estimates the effective number of parameters contributed by both the fixed and random effects [37]. Relative to the null model, the inclusion of useful predictors in a model can increase the shrinkage of the random effects and lead to an overall reduction in effective model complexity (sometimes substantially), despite an increase in the number of included predictors. We then selected a preferred model using a modified one-standard-error rule which mitigates potential overfitting by accounting for estimation uncertainty in the information-theoretic metrics [38]. For the selected model, we calculated the exponentiated coefficients, known as hazard ratios (HR), which provide multiplicative effect sizes for each variable. Survival curves were visualized by separating carcass data into the three community regions (figure 1) and presenting the Kaplan–Meier estimates of the survival function using the packages ‘survival' [39] and ‘survminer' [40].

#### (ii) Carcass use and foraging duration

Carcass use was defined as a binary variable: whether or not a feral cat or forest raven scavenged upon a carcass. Foraging duration was defined as a continuous variable, being the total foraging duration (minutes) by a species at each carcass, calculated by summing the total of all foraging events. A foraging event was defined as consecutive photos of a particular species that was not separated by greater than four minutes of inactivity (i.e. if photos were separated by more than 4 min, there were considered separate events). We visualized the variation in carcass use between the three community regions using the average proportion of carcasses foraged across sites. We calculated the non-parametric bootstrap 95% confidence intervals by performing 10^5^ resamples of the observations for each community region [41]. To analyse the predictors of carcass use by forests ravens and feral cats, we tested a range of *a priori* models based on ecological knowledge (electronic supplementary material, table S4). We tested the effects of five predictor variables: habitat, devil and quoll activity (defined above), plus total foraging duration by devils and quolls (separately, summated minutes). To account for variation in carcass size, we included initial carcass weight as a covariate. We also used site location as a random effect to account for unmodelled variation across the study sites. We again used LOOCV and the modified standard error rule for model selection [38]. We assessed the fit of the top models by calculating the AUC (area under the receiver operator curve; suitable for classification models). We calculated the effect size (ES) for variables within the preferred model by comparing the predicted probability when that categorical variable was applied, against the probability when the effect was absent. Sixteen cameras were removed from this analysis: 12 due to premature removal of the carcass from the field of view and four due to mechanical unreliability or early failure.

To analyse carcass use by forest ravens, we used hurdle models, because the scavenging data were zero-inflated and followed a gamma distribution. We first modelled whether ravens fed at a carcass (GLMMs with binomial link function) and then modelled the total foraging duration by ravens for the carcasses at which they fed (GLMMs with a Gamma distribution and a log link function). Total foraging duration for each camera was calculated by summing the number of minutes any raven spent scavenging on the carcass. GLMMs with a binomial link function were used to assess carcass use by feral cats, but we were unable to model the predictors of total foraging duration by feral cats due to insufficient data, particularly in the full community region.

## 3. Results

### (a) Carcass discovery and persistence

**Figure 2.**
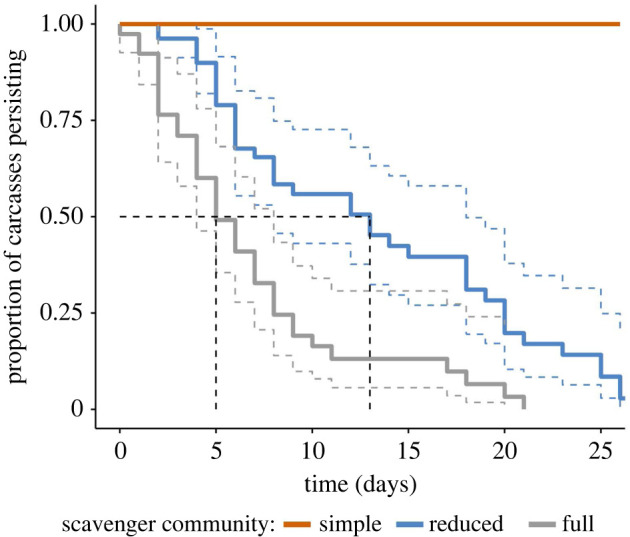
The proportion of carcasses persisting in the environment for each level of scavenger diversity and abundance. Coloured dashed lines indicate the 95% confidence interval. Black dashed line shows the median carcass persistence times for each scavenger community. (Online version in colour.)

Carcasses within the simple community region lasted at least 1.8 times longer than reduced community regions and at least 4.6 times longer than full community region (figure 2). Both devil activity (hazard ratio, hereafter HR = 1.20; 95% confidence interval, hereafter CI: 1.06–1.36) and quoll activity (HR = 1.20; 95% CI: 1.04–1.38) had a negative effect on carcass persistence (see electronic supplementary material, figure S1 and table S2 for model-selection results and electronic supplementary material, table S3 for model output).

**Figure 3.**
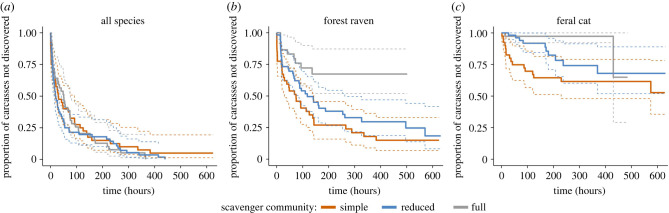
The proportion of carcasses not discovered by (*a*) all species, (*b*) forest ravens and (*c*) feral cats for each level of scavenger diversity and abundance. Coloured dashed lines indicate the 95% confidence interval. (Online version in colour.)

There was no clear difference in the discovery rates between the various scavenger communities when all species were aggregated (figure 3*a*), although carcasses in areas with higher quoll activity were discovered faster (HR = 1.02; 95% CI: 0.99–1.06) while carcasses in wet forests took longer to be discovered (HR = 0.53; 95% CI: 0.37–0.79). Ravens discovered carcasses more quickly in both reduced and simple scavenger communities (figure 3*b*) with devil activity (HR = 0.91; 95% CI: 0.85–0.98) and wet forest habitat (HR = 0.48; 95% CI: 0.24–0.97) suppressing carcass discovery by ravens. Similarly, discovery of carcasses by cats was also suppressed by devil activity (figure 3*c*; HR = 0.88; 95% CI: 0.80–0.98).

### (b) Carcass use and foraging duration

**Figure 4.**
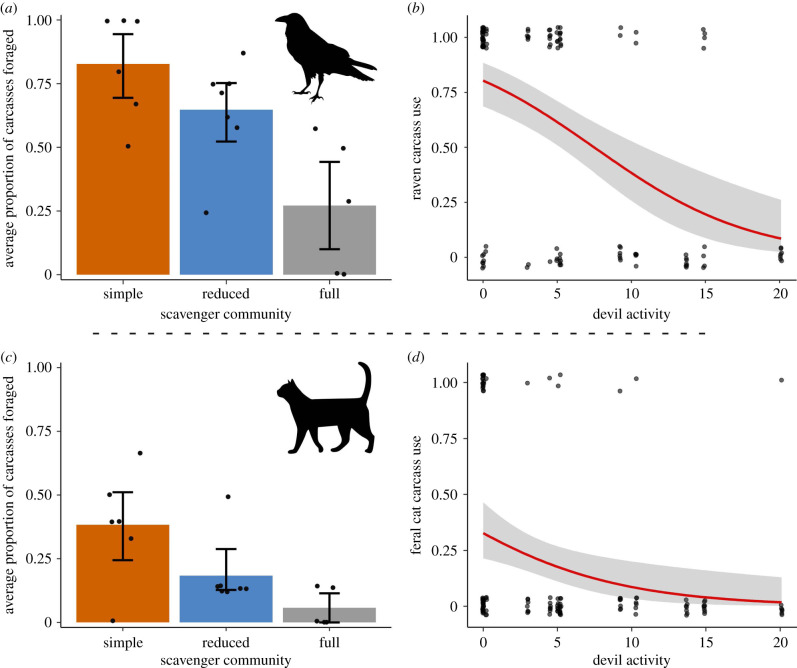
Carcass use by mesoscavengers. (*a*) The proportion of total carcasses foraged by forest ravens, with (*b*) the response curves of the best predictor, devil activity. (*c*) The proportion of total carcasses foraged by feral cats, with (*d*) the response curve of the best predictor, devil activity. In (*a*,*c*), each dot corresponds to the mean value for the study sites and error bars are bootstrapped 95% confidence intervals. (Online version in colour.)

**Figure 5.**
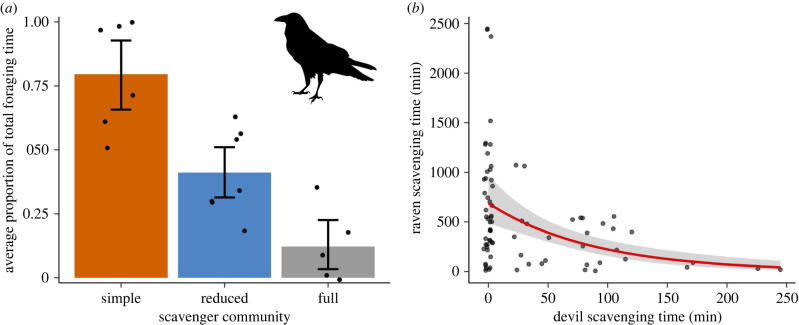
Foraging duration by forest ravens. (*a*) The average proportion of total foraging time, and (*b*) the response curve of the best predictor, devil foraging duration. In (*a*) each dot corresponds to the mean value for the study sites and error bars are bootstrapped 95% confidence intervals. (Online version in colour.)

We recorded 17 species scavenging on the experimentally placed carcasses (electronic supplementary material, table S7). Forest ravens appeared to benefit from the absence of native mammalian carnivores (table 1 and figure 4*a*). As a proportion of total foraging time for all species, we found that ravens fed almost twice as long in the simple scavenger community (88.2% of total foraging time by all species) compared to the reduced scavenger community (47.9%) and five times as long as in the full scavenger community (17.3%; figure 5*a*). Devil activity (ES: 0.97; figure 4*b*) and wet habitat (ES: 0.88) had a negative effect on the probability of a raven feeding at a carcass (see electronic supplementary material, figure S2 and table S5 for model-selection results and electronic supplementary material, table S6 for model output). Devil foraging duration impacted total duration of raven scavenging having an overall negative effect (ES: 0.99; figure 5*b*).

**Table 1.** Number of carcasses foraged, total foraging times and proportion of total foraging time for the top four scavengers (by hours spent foraging) in each scavenger community.

scavenger community (no. carcasses in region)	species	carcasses foraged (% of total)	hours spent foraging (proportion total foraging time per region)
full (40)	tasmanian devil (*Sarcophilus harrisii*)	40 (100%)	108.6 (70.7%)
	forest raven (*Corvus tasmanicus*)	11 (27.5%)	26.5 (17.3%)
	spotted-tailed quoll (*Dasyurus maculatus*)	8 (20%)	8.3 (5.4%)
	feral cat *(Felis catus)*	2 (5%)	4.8 (3.1%)
reduced (56)	forest raven (*Corvus tasmanicus*)	37 (66.1%)	238.7 (47.9%)
	spotted-tailed quoll (*Dasyurus maculatus*)	27 (48.2%)	118.9 (23.9%)
	tasmanian devil (*Sarcophilus harrisii*)	41 (73.2%)	81.2 (16.3%)
	feral cat *(Felis catus)*	10 (18.9%)	23.3 (4.6%)
simple (40)	forest raven (*Corvus tasmanicus*)	32 (80%)	448.3 (88.2%)
	feral cat *(Felis catus)*	16 (40%)	33.9 (7.0%)
	black rat (*Rattus rattus*)	3 (7.9%)	8.7 (1.7%)
	black currawong (*Strepera fuliginosa*)	4 (10.5%)	5.3 (1.0%)

Feral cats scavenged on a higher proportion of carcasses in the absence of native mammalian carnivores. Cats in the simple scavenger community fed on 44% of carcasses, which was 2.4 times more than in the reduced community region (19%), and eight times more than in the full community region (6%; figure 4*c*). The probability of cats scavenging was best predicted by a negative effect of devil activity (ES: 0.89; figure 4*d*).

## 4. Discussion

We used a naturally occurring experiment, of reduction or extirpation of native mammalian scavengers, to examine the effects on scavenging by avian and invasive-mammalian scavengers. This novel scenario, in which mesoscavengers have been allowed to equilibrate to higher resource base (i.e. mesoscavenger release), allows us to test whether smaller species can functionally replace top scavengers. The apex mammalian scavenger, the Tasmanian devil, had an overwhelmingly dominant effect on scavenger dynamics. Smaller scavengers, most notably forest ravens, were the main beneficiary of native mammalian carnivore loss. Further, invasive cats scavenged almost 50% of all carcasses in areas with simplified carnivore communities, highlighting that, contrary to general wisdom [42,43], scavenging is an important source of food for cats [44]. This also suggests potential avenues for reducing the cat's devastating effects on native wildlife [45,46]. Overall, this research highlights the crucial role of scavenging by larger mammals. Rewilding of large carnivores could restore their function within an ecosystem and provide top-down control on mesoscavenger populations [47].

We expected that smaller mesoscavengers would be unable to replicate the scavenging efficiency of the larger, specialist scavenger, the Tasmanian devil [22]. We found that in the region without any native mammalian carnivores, carcasses persisted for almost five times longer than in areas with higher mammalian carnivore diversity (figure 2). While a higher proportion of carcasses in the simple community were large Bennett's wallaby, smaller Tasmanian pademelon carcasses used in the same region also persisted until the end of the study, suggesting comparable patterns of consumption regardless of the initial body size of the carrion. While we chose to use the predominant macropod species within each region as it would reflect the main carrion species that scavengers would feed upon, future studies may choose to use the same macropod species across regions to further ensure the results are standardized. Some carcasses were unexpectedly moved prematurely by forest ravens, feral cats and larger raptors, highlighting the importance of securing carcasses to the ground regardless of prior assumptions. Regardless, these carcasses were located nearby at the conclusion of the study, and none of them had been fully consumed. Previous research has found that mesoscavengers, including corvids, were unable to functionally replace raptors in urban areas, with 70% of fish carcasses remaining [48]. Additionally, the experimental exclusion of carcasses from vultures resulted in 10 times as many carcasses not fully consumed by the remaining scavengers [49]. While all carcasses within the simple community region in our study persisted until the study's end, only 5% of carcasses were not discovered and eaten by the remaining mesoscavengers (figure 3*a*). Seemingly, the absence of native mammalian carnivores had little impact on the remaining scavengers locating and feeding upon the carcasses. This indicates little facilitation of carrion resources by top scavengers through advertising or increased accessibility in our study system.

We found evidence that top carnivores limit carrion access for smaller scavengers. Ravens were the main beneficiary of native mammalian carnivore loss, with ravens in the simple community region finding 88% of the carcasses (figure 3*b*) and foraging for seven times longer than in the full scavenger community regions (figure 5*a*). Devils suppressed raven carcass utilization (figure 5*b*), probably because nocturnal devils consumed the resources before diurnal ravens discovered or fully used them. This finding supports previous evidence that under low levels of competition raven populations on the Bass Strait islands prioritize scavenging on carrion, such as roadkill, across the entire year even when other resources (e.g. invertebrates, fruit, seeds) are available [50]. Both raven carcass use and carcass discovery by all species was reduced in wet forests, likely due to habitat preferences or reduced visibility in these environments.

Until recently, cats were believed to rarely scavenge [42,43]. However, there is now a growing body of evidence that they actively scavenge, especially when they perceive little risk [44]. Our data show that in the simple scavenger community, feral cats scavenged at eight times the rate of cats living in a full scavenger community (figure 4*c*). This supports work on the stomach contents of feral cats on King Island, which found that most of their prey items were pademelons and wallabies which, given their size, were most likely scavenged [51]. Furthermore, the presence of devils in the landscape appeared to supress cat scavenging behaviour, potentially through interference competition (cats also being mostly nocturnal foragers). Past research has demonstrated that the presence of devils within an environment can trigger avoidance strategies in cats to evade interspecific conflict [15,52]. Reduced interference competition caused by top-carnivore loss can have cascading effects throughout an ecosystem, potentially leading to population increases of, and expanded functional roles for, smaller carnivores [53]. However, fear effects imposed by larger carnivores on mesocarnivores are not fully understood, and further studies are required to disentangle these dynamics between carnivores [19]. Combining several lines of investigation (e.g. GPS data on multiple predators combined with cameras on carcasses) would help quantify the risk-reward trade-off of carcasses, and could help reveal under what circumstances carcasses are ‘fatally attractive' to mesopredators [20,54].

Following the disease-driven decline of devils across Tasmania, quolls also increased their use of carrion in areas of low devil density [15]. Indeed, in areas of greater devil decline, such as north-Eastern Tasmania, quoll scats contained many large-mammal remains, suggesting that the loss of top scavengers improved scavenging opportunities for quolls [55]. Despite quolls being mesoscavengers, they have, like devils, also been documented chasing cats from carcasses, providing evidence of interference competition [15]. While carcasses persisted in the absence of quolls (figure 2), we only found a weak effect of quoll abundance on carcass use by ravens and cats. As quolls are non-specialized and smaller scavengers, they are much less efficient than devils and it is therefore difficult for them to monopolize a carcass in the same way [56]. Additionally, the effects of devils—as a dominant and specialized scavenger—on other opportunistic scavengers might simply be too strong, acting to mask any potential impacts the quoll may have on cats and ravens [22].

We should note that the reported effect estimates and their confidence intervals for all analyses were taken from the model selected by cross validation, yet it is known that post-selection inference on parameter estimates can be biased due to failure to account for model-selection uncertainty [57]. To check the validity of these inferences, we compared the estimates to those of the full model which is known to closely approximate valid post-selection inferences [58]. We found the size of the estimates were similar for all of the analysis, with the exception of the carcass persistence models for which the devil and quoll activity had slightly lower estimates but lead to the same conclusions.

As highly efficient scavengers, the loss of apex scavengers can lead to increased food availability for mesoscavengers which could result in increases in abundance [1]. For example, the absence of vultures (*Gyps* spp.) in southeastern Spain led to a higher abundance of red foxes (*Vulpes vulpes*) due to greater availability of carrion [59]. In the Bass Strait region, anecdotal evidence suggests that forest raven and feral cat populations are growing on King Island [27]. Enhanced opportunities to feed to on roadkill [50] and other carrion, as demonstrated in this study, may provide explanations for this apparent increase in abundance. Further research is needed to confirm whether these species are truly increasing in abundance. Elevated numbers of forest ravens could have destructive effects for the local birds on the islands through heightened levels of depredation, and impact local farmers through increased attacks on livestock, as shown in other corvid studies [44,60], as well as on King Island specifically [61]. While past research in Tasmania found no impact of forest raven abundance on the abundance of other bird species [62], these impacts may differ on the Bass Strait islands if the raven population size is greater. Meanwhile, the impacts of the invasive feral cat on small mammals or birds are well documented, with many species now threatened with extinction or already lost due to heightened predation risk [63,64]. Despite these apparent increases in abundance, feral cats and forests ravens are less efficient scavengers than devils [56], meaning carcasses may persist in an environment for longer. This could have adverse effects on both animal and human health due to the increased spread of carrion-borne diseases [1,18].

Large carnivore populations have fluctuated due to human persecution and habitat loss, causing trophic cascades throughout food webs across the globe [6]. In our study, we found that top scavengers, like Tasmanian devils, limit carrion use and discovery by smaller scavengers, such as ravens and cats. However, it remains unclear how this may impact mesoscavenger population abundance and whether there are cascading effects on small prey species. In the absence of top mammalian scavengers, we found that carcasses persisted beyond the study length (approx. three weeks). Further research is required to see how this may impact the transmission of carrion-borne diseases and scavenging by invertebrates, which was not monitored because the study was done in winter. While earlier work has found that devil declines were associated with an increase in carrion-persistence times and access for smaller scavengers [15], here we demonstrate that the complete loss of top mammalian scavengers further magnifies those impacts. Thus, it seems that even low densities of native large carnivores can fulfil at least some of their usual functions. This suggests that restoring native carnivores, even to low densities, can provide some benefits, providing support for novel management approaches such as trophic rewilding [47,65]. Overall, our findings further highlight and clarify the integral role native mammalian scavengers perform within an ecosystem, demonstrating the ecological significance of global mammalian carnivore conservation.

## Data Availability

Data are available from the Dryad Digital Repository: https://doi.org/10.5061/dryad.ghx3ffbrf [66]. Code is available from the Zenodo Repository: http://doi.org/10.5281/zenodo.6629940 [67]. Electronic supplementary material is available through Figshare [68].
